# Medical malpractice in organ transplantation: public allegations and key legal outcomes

**DOI:** 10.3389/frhs.2024.1408934

**Published:** 2024-08-12

**Authors:** Panos Arvanitis, Michel R. Davis, Abby London, Dimitrios Farmakiotis

**Affiliations:** ^1^ Division of Infectious Diseases, Warren Alpert Medical School, Brown University, Providence, United States; ^2^ Warren Alpert Medical School, Brown University, Providence, United States

**Keywords:** malpractice litigation, medical negligence, organ transplantation, patient safety, litigation outcomes

## Abstract

**Introduction:**

Despite significant advances in surgical techniques and patient outcomes, organ transplantation (OT) remains fraught with legal challenges and ethical dilemmas. This study aims to address the notable gap in literature on malpractice claims specifically related to OT, providing insights into litigation trends, outcomes, and implications for medical practice and patient care.

**Methods:**

We retrospectively queried the Verdictsearch database from 1988 to 2023, and captured malpractice claims involving several organs. Data on demographics, organ types, and litigation outcomes were collected to compare compensation across different categories of malpractice and patient outcomes.

**Results:**

Out of 292 malpractice cases identified, 62 met inclusion criteria, distributed across 19 states with kidney being the most implicated organ (46.8%). Defendants prevailed in 53.2% of cases, while settlements were reached in 29.0%, and plaintiffs won in 16.1% of cases. Surgical errors and complications were the most frequent allegations, followed by medication and treatment errors. The median compensation for deceased plaintiffs was significantly higher ($1,300,000) compared to living plaintiffs at litigation initiation ($128,000).

**Discussion:**

Our study sheds light on the challenges and trends in malpractice litigation within the field of OT. By identifying key areas of concern and the influence of patient outcomes on litigation resolution, this study offers valuable insights for healthcare providers, legal practitioners, and policymakers aimed at enhancing patient safety, reducing litigation risks, and fostering a deeper understanding of the ethical and legal complexities in OT.

## Introduction

Malpractice litigation is common in the field of surgery. According to the American Medical Association, a staggering 66.3% of surgeons aged 55 and older have encountered at least one lawsuit during their career (1). While surgical errors contribute to a significant portion of the 85,000 medical malpractice claims filed annually in the U.S., there is a notable absence of literature focusing on malpractice specific to organ transplantation (OT) (2). Addressing this gap is essential for patient safety, upholding medical standards, and guiding best practices.

A pivotal case, Good v. Presbyterian Hospital, serves as an introduction to the complexities of malpractice litigation in OT, emphasizing the necessity for medical practitioners to possess a keen understanding of the legal implications of their practices. This case revolved around a critical issue of informed consent, where the plaintiffs alleged that the failure to disclose the donor organs' cytomegalovirus (CMV) status contributed to the adverse outcome of a heart and lung transplant. The court's decision to side with the defendants, based on the argument that disclosing the CMV status was not standard practice, underscores the necessity of aligning with established medical standards and the expectations of the legal system regarding patient care. Similarly, the RaDonda Vaught case underscores the significance of systems thinking in healthcare. Vaught's conviction for criminally negligent homicide, following a fatal medication error, highlights systemic failures in the healthcare system. Both cases signal a pressing shift towards a legally informed and systemic approach in medical practice, underlining the intersection of medical actions and legal accountability.

As the field of OT continues its growth and evolution, detailed knowledge of malpractice claims could help improve patient care and foster greater trust in this essential medical intervention, by offering insights into best practices in pre-transplant evaluation, surgical techniques and complications, and the potential legal consequences of adverse incidents.

To our knowledge, no previous studies have focused specifically on malpractice claims involving OT candidates or recipients (OTR). To this end, this study aims to explore the nature and frequency of malpractice claims in OT, given the unique complexities and high-stakes nature of transplantation procedures. We hypothesized that the nature of litigation in OT might present distinctive patterns due to the critical and intricate nature of these surgeries.

## Methods

Patients who either underwent OT or were registered on the waiting list and had their cases indexed by the Verdictsearch database (ALM Media Properties, LLC, New York, NY) qualified for inclusion in our study. This database is among the most extensive in the US for case information and has been utilized in numerous publications that investigated medical malpractice across various medical disciplines (3–7). PA and MRD were responsible for the manual review and screening of the cases. Discrepancies during this process were resolved by AL and DF.

Our search, spanning from 1 January 1988 to 31 December 2023, focused on malpractice in OT, using Boolean operators to refine our inquiry. Keywords included “medical malpractice” combined with AND/OR “transplant,” “organ,” “liver,” “heart,” “kidney, “pancreas”, “bowel” and the terms “organ transplantation”, “transplantation”, and “solid organ transplantation” to widen our scope. We also queried “donor complications” with “litigation,” “informed consent” with “transplant outcomes,” and “surgical errors” or “negligent care” related to transplantation. Exclusion criteria were: (i) bone marrow transplantation, (ii) claims unrelated to the transplanted organs, and (iii) OTR suing for OT-unrelated reasons.

We collected data related to patient demographics and details about the type and number of organs transplanted. The outcomes of interest were on litigation resolution: Specifically, outcomes included verdicts, differentiating between those in favor of the plaintiff and those in favor of the defendant, mixed verdicts, settlements, and arbitrations, as well as the amounts of monetary awards. Cases settled before court proceedings or formal registration are not captured in Verdictsearch. Therefore, settlements herein refer to post trial registration and/or court proceedings agreements made outside of trial between the patient and the physician/hospital organization. The Verdictsearch database defines mixed verdicts as judgments against the physician that differ from the initial claims.

**Figure 1:**
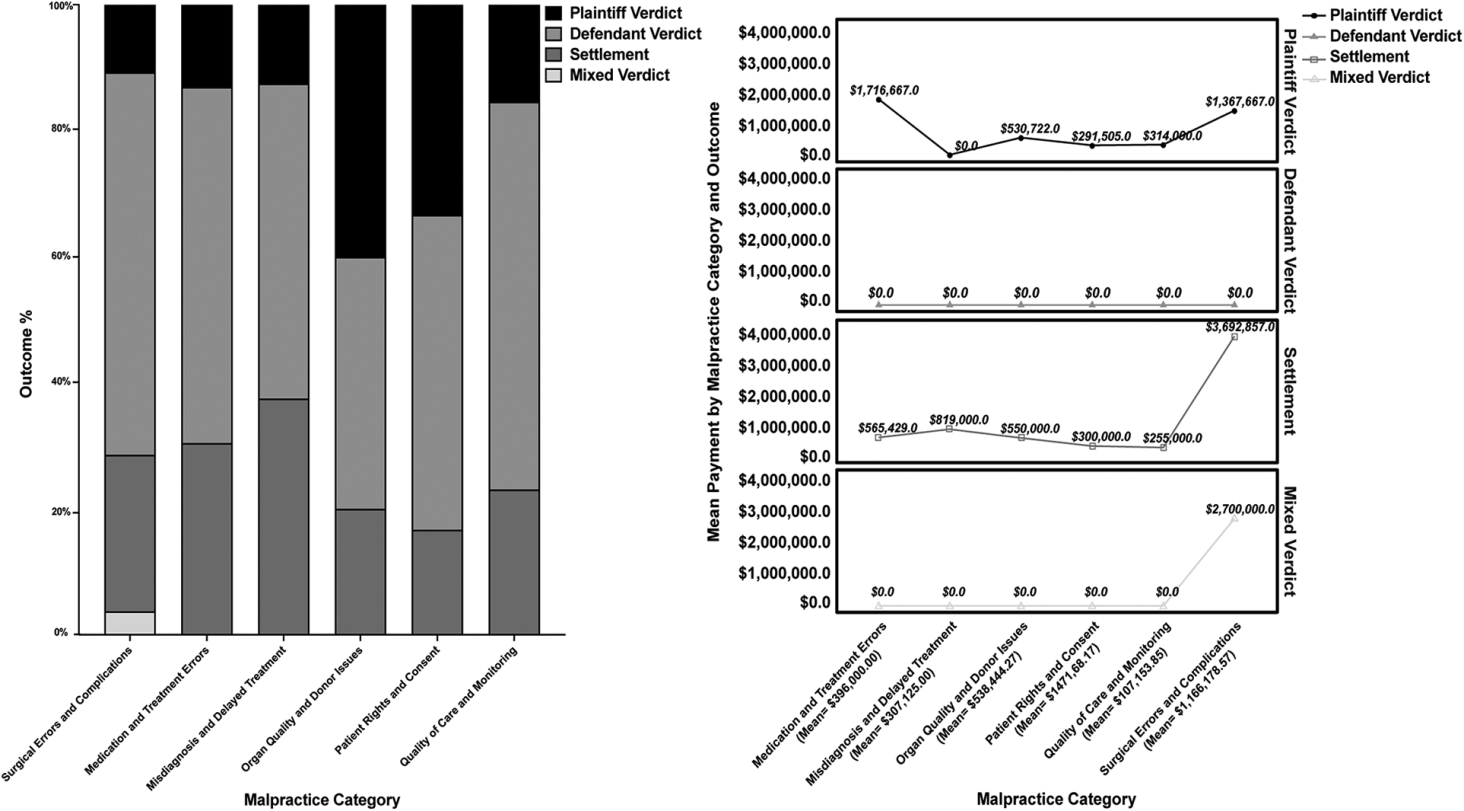
Malpractice categories by outcomes and mean payments by malpractice category and outcome.

Cases were then categorized based on aspects of medical malpractice, encompassing six categories-domains: surgical errors, medication and treatment errors, misdiagnosis and treatment delays, concerns regarding organ quality and donor-related complications, violations of OTR patient rights and informed consent, and broader concerns about the quality of care and monitoring (Table 1 and Figure 1). These categories highlight key aspects of malpractice cases relevant to study outcomes. Within the broad scope of medical malpractice, it is possible for the variety of categories to exceed the number of legal outcomes, as some cases have multiple components. We describe characteristic cases from each category.

**Table 1:** Legal outcomes and malpractice categories.

**Legal outcomes (*N* = 62) (%)**	
Plaintiff verdict	10 (16.1)
Defendant verdict	33 (52.2)
Settlement	18 (29.0)
Arbitration	0 (0)
Mixed Verdict	1 (1.6)
**Medical malpractice categories (*N* = 88) (%)**	
Surgical errors and complications	28 (31.8)
Intraoperative	13 (14.8)
Postoperative	15 (17.0)
Medication and treatment errors	23 (26.1)
Misdiagnosis and delayed treatment	8 (9.1)
Organ quality and donor issues	10 (11.4)
Patient rights and consent	6 (6.8)
Quality of care and monitoring	13 (14.8)

Continuous data are primarily presented as median (IQR). The Wilcoxon-Mann-Whitney test is employed to compare the distributions of mean payments between two distinct groups—those labeled “deceased” and “alive”. For a broader analysis across more categories, we used the Kruskal-Wallis H to identify significant differences in median payments, with subsequent pairwise comparisons using the Mann-Whitney *U*-test when significant disparities were detected. For specific analyses on malpractice categories' payments, we complement this with mean payments (±SD) to highlight both average and exceptional cases (“outliers”) within each category.

This research did not require Institutional Review Board approval since the database is publicly accessible. All statistical analyses were conducted using R, version 4.0.5 (R Foundation for Statistical Computing). A two-tailed *p*-value of 0.05 determined statistical significance, unless stated otherwise.

## Results

**Figure 2:**
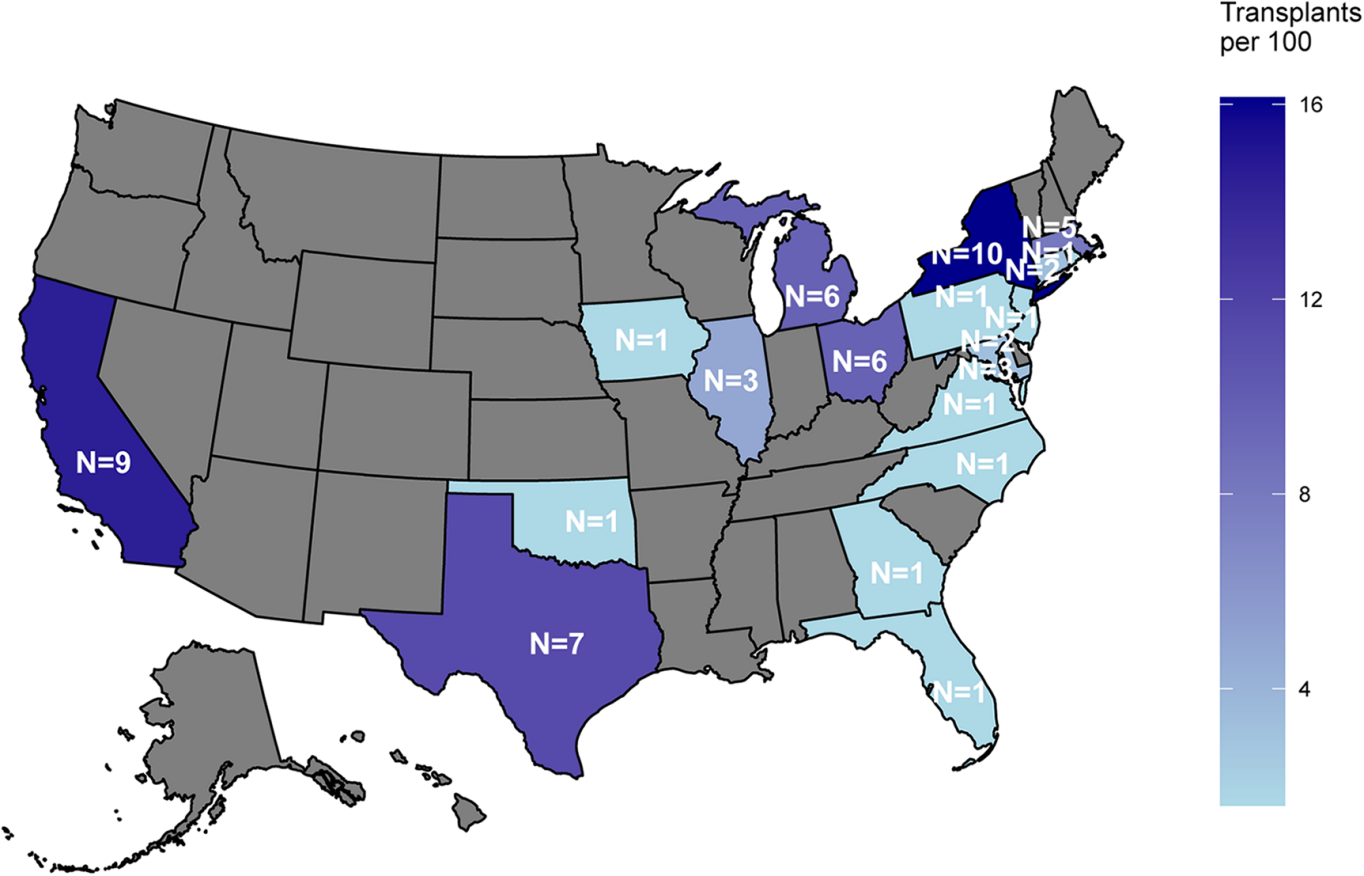
Distribution of litigation by state.

Our initial search identified 292 malpractice cases. Of these, 79 were pertinent to our research topic; the excluded cases either did not concern the endpoints of interest, involved only peripheral discussions of malpractice, or pertained to unresolved legal consequences. From the relevant set of 79, 62 cases met inclusion criteria. These cases were identified across 19 states, which represent the entirety of states where cases were documented within the database, for the scope of our study. Cases were most frequently identified in New York (16.1%, 10/62), closely followed by California (14.5%, 9/62), and Texas (11.3%, 7/62), reflecting transplant “density” (Figure 2). Across the 62 cases, a total of 64 organs were transplanted. Most defendants were male (64.5%, 40/62), with a median age of 46 (29–56) years. By the time litigation took place, 69.4% (43/62) of the plaintiffs had passed away and were represented by their family's estates (Table 2). Kidney was the most frequently transplanted organ, accounting for 46.8% (30/64) of all organs, followed by liver (28.1%, 18/64). Race and ethnicity data were not available in the database.

**Table 2:** Baseline characteristics.

Total lawsuits	79 (100%)
Meeting inclusion criteria	62
Not meeting criteria	17
Number of states represented	19
**Sex (%)**	
Male	40 (64.5)
Female	22 (35.5)
Age (Median, IQR)	46 (28.8–56)
**Present health condition of patients (%)**	
Dead	43 (69.4%)
Alive	19 (30.6)
**Transplantation status (%)**	
Transplanted (previously received)	47 (75.8)
Transplant awaiting	15 (24.2)
**Transplant organ details (%)**	
Kidney	30 (48.4)
Liver	18 (29.0)
Heart	12 (19.4)
Intestines	1 (1.6)
Thymus	1 (1.6)
Lung	2 (3.2)
Multiple organ transplants (%)	2 (3.2)

Data are presented as number (percentage) for categorical variables and median [interquartile range (IQR)] for continuous variables. All patients were coded as either female or male in the EMR; none were listed as intersex.

In terms of case outcomes, defendant verdicts prevailed in 53.2% (33/62), followed by settlements (29.0%, 18/62). Plaintiff verdicts were observed in 16.1% (10/62), with a single mixed case. Notably, no arbitrations were identified so they were not included in further analysis (Table 1). In our analysis of medical malpractice, we identified 88 different allegations across the six categories. Allegations can be representative of various malpractice categories, thereby accounting for the number of allegations exceeding the number of cases (Table 1 and Figure 1).

**Figure 3:**
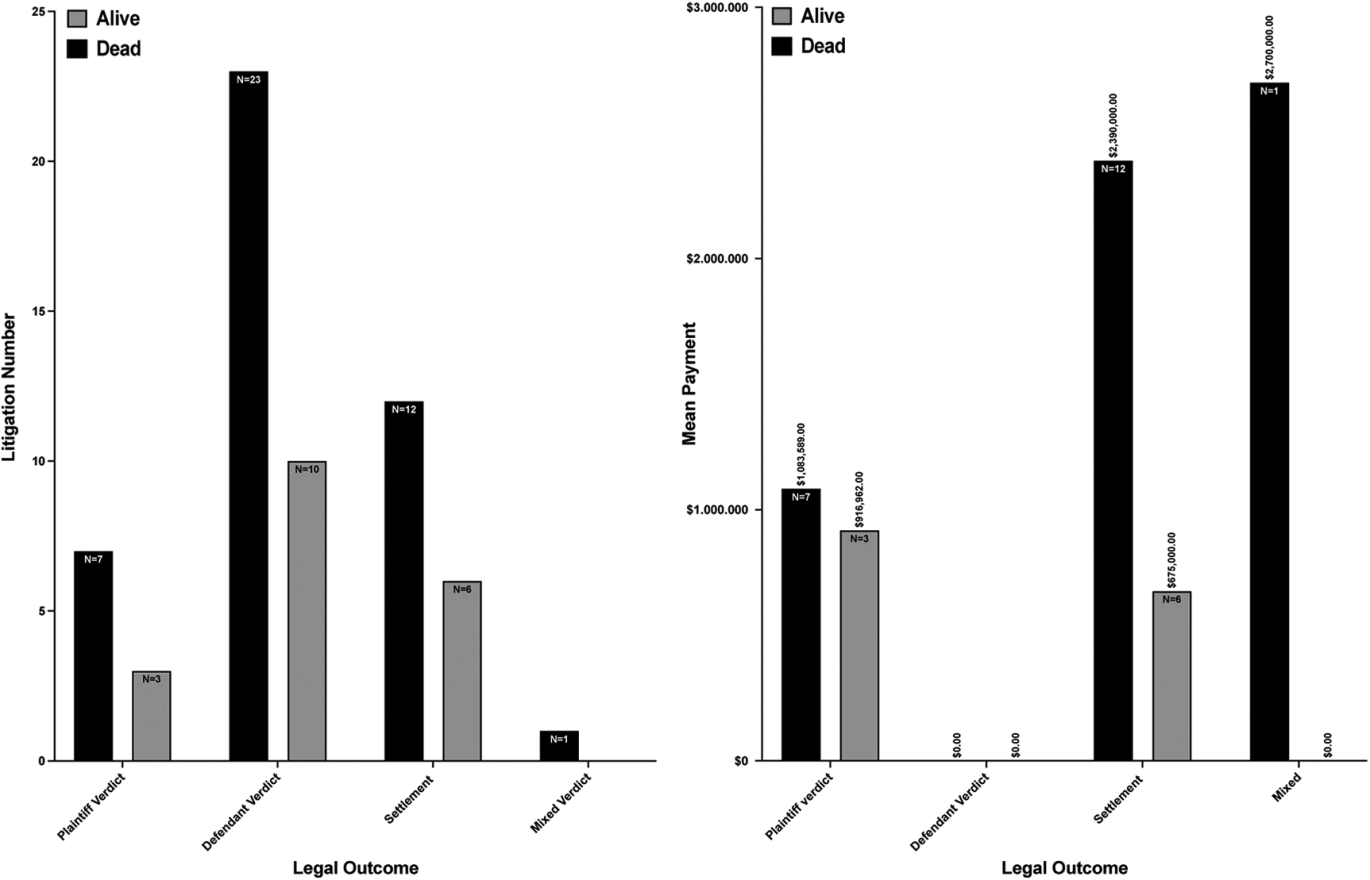
Litigation number and mean payment by legal outcome.

Regarding deceased or alive status of the plaintiff at the initiation of litigation (Figure 3), a substantial contrast in compensation amounts was evident between these two categories. For cases resulting in a plaintiff's verdict, individuals alive at the start of litigation had a median compensation of $128,000 ($122,887-$2,500,000). In contrast, deceased plaintiffs had a median payment of $1,300,000 ($460,122-$1,500,000) (*p* = 0.004). In settlement scenarios, deceased claimants were awarded a median of $607,500 ($317,750-$1,637,500), higher than the median of $475,000 ($75,000-$1,500,000) for those still alive at the initiation of litigation (*p* < 0.0001). Compensation in one case with a mixed verdict reached $2,700,000.

Highlighting specific cases, the largest settlement of $19,900,000 arose from an intraoperative error during a heart transplant in Ohio, resulting in the patient's death. This error involved a failure to clamp the aorta, leading to hemorrhagic shock. Conversely, the smallest settlement, also in Ohio, amounted to $100,000 due to a foreign object (a suction cylinder) being left in a patient after a liver transplant.

Regarding different categories of malpractice, surgical errors and complications were the most common reason for litigation in 28 cases (31.8%). Within this category, intraoperative and postoperative issues were almost evenly distributed, with 13 and 15 cases, respectively. When compensation was awarded, the average amount was $1,166,178.57 (±$3,694,495.33), with a median of $0 ($0-$1,250,000). Medication and treatment errors followed with 23 cases (26.1%), with the average compensation being $396,000.00 (±651,064.21), and the median $0 ($0-$750,000). Misdiagnosis and delayed treatment comprised 8 cases (9.1%), with an average reparation of $307,125.00 (±$551,378.15) and a median of $0 ($0-$414,250). Issues surrounding organ quality and donor-related complications were found in 10 cases (11.4%), with an average compensation of $538,444.28 (±$814,692.17) and a median of $122,887 ($0-$750,000). Matters of patient rights and informed consent were less frequent with only 6 cases (6.8%) and had average payments of $147,168.17 (±176,476.00) with a median of $61,444 ($0-$340,031). Lastly, quality of care and monitoring involved 12 cases (13.6%), with an average compensation of $107,153.85 (±$180,457.47) and a median of $0 ($0-$214,000). The Kruskal-Wallis H test indicated no significant differences in compensation across the various categories of malpractice (*p* = 0.844), likely due to the small number of cases in most domains and large variations.

Surgical Errors and Complications were further categorized into intraoperative (13/28) and postoperative (15/18) errors. Within the intraoperative group, surgical mistakes accounted for 69.2% (9/13), encompassing issues like incorrect tube insertion, improper clamping, transplantation of mismatched organ sizes, and unintentional retention of foreign objects such as sponges or stents. Another 30.8% (4/13) of these cases pointed to inadequate organ inspection and selection, which led to oversights like missed flaws in donor hearts or unintentional disposal of usable kidneys.

Acute transplant rejection was evident in 33.3% (5/15) of post-operative complications, in kidney, liver, and heart transplants. One error was the administration of a nephrotoxic drug, gentamycin, causing immediate kidney failure. One patient experienced a fall post-surgery. Additional immediate post-transplant complications included post-surgical bleeding, organ dysfunction, and complications due to inadequate hydration.

When assessing medication and treatment errors, 43.5% (10 out of 23) were related to errors in medication administration. The primary issues involved healthcare professionals' lapses in ensuring accurate medication delivery, mismanagement, and lack of monitoring, resulting in events such as subsequent transplant rejection or adverse drug reactions. The remaining cases highlighted treatment errors, including mislabeling, such as incorrectly labeling a patient as “post-heart transplant” when they were actually “pre-surgery”.

Half of the misdiagnosis and delayed treatment cases (4/8) were associated with postponed care. One example involved a heart transplant recipient being prematurely discharged, without adequate monitoring or follow-up care, which could have contributed to subsequent fatal congestive heart failure initially misdiagnosed as typical chest pain expected as part of the recovery process after heart surgery. Other cases in this section overlapped with different categories. A notable instance concerning delayed treatment was a patient delisted from a kidney transplant list due to procrastinated foot ulcer treatment, which led to infection and prolonged hospitalization, ultimately causing him to become ineligible for further consideration for the transplant. Regarding organ quality and donor issues, one involved a heart donor with evident atherosclerosis and history of intravenous drug use, with the recipient dying. Another case highlighted a “high-risk” kidney transplant from a donor with an unclear background, including homelessness, undisclosed sexual history, and intravenous drug use, potentially leading to a subsequent Lymphocytic Chorio-Meningitis Virus infection in the recipient. This caused the patient to require dialysis post-transplant, significantly impacting their quality of life. In another case, a transplanted kidney identified as having cancer had to be surgically removed.

In the domain of patient rights and consent, one case recounted a kidney recipient facing kidney failure and asserting lack of informed consent. Another incident involved a liver transplant recipient contracting hepatitis B due to a non-disclosed high-risk organ donor. In one case, linguistic barrier resulted in a patient unwittingly accepting a high-risk kidney.

Finally, under the quality of care and monitoring category, one case highlighted a patient's difficulty transitioning off a bypass machine, attributable to the non-availability of qualified doctors. Another claim detailed a 19-year-old's death from brain injury post-kidney transplant, linked to alleged lapses in monitoring and response. Another case involved a lung transplant candidate, who was not transplanted due to insurance-based referral refusals, which purportedly led to their demise.

## Discussion

Celebrating a significant milestone, the US recently acknowledged surpassing one million organ transplants by September 9, 2022, marking a significant advancement in organ donation, and further highlighted by exceeding 40,000 transplants in 2022 alone (8). As a result of this trend, it is reasonable to anticipate a corresponding surge in malpractice claims associated with transplant procedures. This underscores the importance of emphasizing quality care, fostering effective communication between patients and healthcare professionals to preemptively address potential issues, and engaging more healthcare professionals in discussions surrounding this issue to ensure a comprehensive approach to patient safety and care. OT is distinguished from other medical fields by its complex ethical and clinical decisions, made necessary by the limited availability of organs and the critical condition of most recipients. Transplant teams often face urgent judgment calls with incomplete information about donor comorbidities, which significantly complicates the landscape of medical litigation. Moreover, the outcomes of OTs hinge on the quality and compatibility of the donor organ, introducing a unique level of risk and ethical responsibility. Decisions about donor organ selection and informed consent are typically made amidst uncertainty, based on partial donor medical histories and urgent clinical needs. The clear communication of potential risks, such as potential organ dysfunction and disease transmission, is crucial. These risks must be clearly explained to recipients, who may vary in their risk tolerance depending on their condition and the type of organ needed. For instance, recipients in urgent need of heart, liver, or lung transplants may be more willing to accept substantial risks compared to kidney recipients, who might consider dialysis which is a less risky alternative. Additionally, these differences in risk perception and acceptance can profoundly influence legal outcomes, with courts often considering the urgency of the recipient's condition, the potential for alternative treatments, and the established medical and ethical guidelines when evaluating cases involving organ transplant decisions.

Data regarding malpractice in OT is notably limited in the literature. Holman et al. investigated medical malpractice allegations in hepatology from 2012 to 2021, incorporating allegations related to liver transplantation from their local center and a comprehensive national insurance database (9). They identified 11 different kinds of transplant allegations: 4 pertained to perioperative morbidity, 3 to intraoperative morbidity, and the remainder addressed biopsy errors and falls. Unlike our study, the specific nature of the litigation, whether it was in favor of the plaintiff or defendant, and payment amounts were not detailed in their findings (9). Importantly, the national database, encompassing records from over 550 US hospitals, revealed 94 medical malpractice claims related to hepatology from a total of 102,575 claims. The bulk of these claims were attributed to diagnostic errors (56%), miscommunication (22%), and patient behavior issues (20%), yet none were directly relevant to liver transplantation. Despite the relatively small number of cases reviewed, our study addresses an important gap in the literature, providing novel findings with potentially significant implications for clinical care and healthcare policies.

In more than half (53.2%) of cases in our study, defendant verdicts prevailed. Several factors, commonly encountered in such trials, contribute to this predominance: Many of these defending entities possess well-established legal teams that specialize in medical malpractice defense, enabling them to mount a substantial defense. The intricate nature of medical cases often benefits the defense, who can argue that complications or adverse outcomes are inherent risks associated with the procedure rather than stemming from negligence. Consequently, the responsibility frequently lies with the plaintiff to demonstrate negligence, a significant challenge due to the sophisticated nuances of medical care. Moreover, while plaintiffs often hire malpractice lawyers who utilize expert witnesses to challenge the care provided, defendants typically engage credible expert witnesses who can attest that the administered care adhered to standard medical practices, reflecting the healthcare system's capacity to assemble a more robust and comprehensive legal representation The general public sentiment might also lean towards medical professionals, with juries frequently sympathizing with them, believing that they executed their duties to the best of their capabilities given the circumstances. A lack of strong evidence of negligence from the plaintiff, combined with the defense introducing arguments related to comparative or contributory negligence, can further tilt the case in the defendant's favor. All of the above likely support the defendants even more in the field of transplantation, given its complexity, the altruistic nature of organ donation, and overall implications for society.

The cases of Good v. Presbyterian Hospital and RaDonda Vaught highlight critical aspects of malpractice litigation. The Good v. Presbyterian Hospital case illustrates the complexities of informed consent and aligning medical practice with legal standards. In this case, the recipient contracted CMV and subsequently died due to complications from the infection, having received an organ from a CMV-positive donor while being CMV-negative themselves (D+/R-). The court's decision in favor of the defendants, based on the non-standard disclosure of CMV status, underscores the importance of adhering closely to established guidelines to mitigate legal risks. This case highlights the need for clear communication protocols and standardized practices in transplant medicine, especially regarding the disclosure of donor organ conditions that may significantly impact patient outcomes. This is particularly pertinent in OTs, as evidenced by Good's case, where informed consent regarding donor organ conditions significantly impacts outcomes for OTRs. Conversely, the RaDonda Vaught case highlights the significance of systems thinking in healthcare. The case of Vaught, who was convicted of criminally negligent homicide due to a medication error where she administered vecuronium instead of Versed® (midazolam), resulting in the patient's death, sparked widespread debate in the healthcare community about the criminalization of medical errors and its potential impact on patient safety reporting. Eventually, Vaught's conviction revealed systemic vulnerabilities. This case emphasizes the need for comprehensive safety protocols and institutional accountability, underlining the importance of fostering a culture of safety and continuous improvement within healthcare systems. The contrasting outcomes of these cases—with Vaught facing criminal charges while systemic issues at her hospital went unaddressed—raise questions about equitable accountability in healthcare and the potential effect on error reporting. In OT, this underscores the necessity of stringent safety measures and the potential repercussions of systemic failures on OTR. The cases highlight the broader implications of malpractice litigation, emphasizing the importance of patient safety, effective communication, and systemic resilience in healthcare practices. Additionally, while these cases provide insights, they do not set a new legal precedent. This absence of precedent-setting cases in our study may suggest generally favorable outcomes in transplant-related litigation, indicating that existing legal frameworks and medical standards adequately address most issues encountered in such cases.

Almost all transplant candidates and recipients forge a deep relationship with their care team, a factor that likely further decreases the inclination to sue even when complications arise. This premise is supported by our observations: the low number of litigations in our study can be largely attributed to the robust relationship between patients and their multidisciplinary transplant team providers (10, 11). Many transplant centers have introduced a “working network” of professionals, focused on holistically addressing factors impacting patient health—from social and environmental to psychological and genetic determinants—while also aiding in the re-employment, family, and social reintegration of transplant patients (12–14). These teams provide comprehensive support throughout the transplant journey, fostering a deep-seated trust between patients and providers (15). Additionally, several centers have inaugurated routine meetings with representative subsets of the team to discuss patient cases (13). The culmination of these integrated efforts likely explains the scarcity in litigation and, consequently, the limited literature on transplant malpractice, highlighting the effectiveness of patient-centric models in transplant medicine.

We found that the representatives of deceased individuals often secured larger monetary settlements, based on the difference in payments between “alive” and “deceased” plaintiffs. This disparity can be attributed to a variety of reasons: Death might be perceived as the most severe consequence of medical negligence, thereby justifying heftier settlements compared to non-fatal outcomes (16, 17). The death of a patient can evoke stronger emotional reactions from jurors, making them more sympathetic towards the deceased's family, possibly influencing higher compensation awards (18). Settlements might also take into consideration the entirety of the deceased's potential future earnings, thus increasing the compensation amounts (19). Lastly, the representatives of deceased individuals, often their families, might pursue cases with greater vigor, fueled by a desire for justice for their loss (18, 20). It should be noted that transplant surgeries are, as a rule, performed on individuals expected to survive, who have a reasonable long-term prognosis after transplantation; therefore, the death of a transplant recipient raises often the question of a medical error, and signifies loss of income, resulting in marked financial distress for their families.

Notably, Palaniappan et al. conducted a retrospective study focusing exclusively on heart transplantation and identified 41 cases of interest (21). Their research also highlighted differences in award sizes based on survival status after the procedure. However, they found that survivors received larger awards due to anticipated future care costs or claims for lost productivity (21). Contrary to their findings, our study demonstrated that representatives of deceased individuals often secured higher compensations. This discrepancy suggests a possible variation in the factors influencing awards across different transplantation types, or changes in the legal landscape over time.

The analysis within our study elucidates a significant trend: kidney transplants, which constituted 46.8% of the organ transplants in our dataset, had the most malpractice claims. This trend is consistent with national transplant statistics, with a record set of over 25,000 kidney transplants performed in 2022, paralleled by a network of 256 adult kidney transplant centers nationwide (22). This expansion in both procedures and facilities indicates a response to the escalating demand for kidney transplants and likely contributed to the higher incidence of related malpractice claims, as observed in our analysis.

The Verdictsearch database, while informative for malpractice cases, has its limitations. Data were obtained retrospectively, yet we ensured solid definitions for outcome extraction. The US focus of this study limits its applicability to transplant centers in other parts of the world, which may operate under different legal frameworks and practices, underscoring the need for more studies in this field. Also, the database's scope is limited; it only contains malpractice suits from states where cases are documented and shared resulting in a regionally biased perspective. The recorded litigation from 1988 to 2019 might not reflect the full spectrum of cases within the U.S. during this period and, most importantly, the payment amounts entered may be inaccurate due to reporting or transcription discrepancies. One additional limitation is our reliance on a single database for case identification. This database is most used in the US for medical malpractice cases and several studies have employed it for similar research purposes (3–7). Unlike systematic literature reviews in medicine, where multiple databases are typically queried to ensure comprehensive coverage, our study's focus on legal case data restricted us to this legal database. The use of only one database might limit the breadth of cases captured and could potentially introduce selection bias. While there are other legal databases such as LexisNexis and Westlaw that could be queried, the inherent difficulties of extracting and comparing case data from multiple databases pose significant challenges (23). Many of these databases are not publicly accessible, requiring specific subscriptions or affiliations, which can limit availability. Additionally, and most importantly, each database often employs unique classification systems and terminology for cataloging cases, leading to a lack of standardization that complicates comparability. The entry formats can also differ significantly, with some databases providing extensive narrative descriptions of the cases and others offering only summaries. A futher limitation of our study is the lack of detailed information on how established clinical standards influenced individual cases, as these were not recorded or examined in the database. Additionally, the varying levels of evidence supporting guidelines complicate their role in legal outcomes and how they are recorded or interpreted in court. Some guidelines are based on expert consensus rather than high-quality evidence, leading to challenges in interpretation during legal proceedings. This discrepancy can affect the determination of the standard of care, highlighting the need for more research, and potentially clearer guidelines to ensure consistent application in malpractice cases.

Another significant limitation is that a large majority of cases are settled prior to court and not recorded in Verdictsearch. Importantly, key plaintiff details, such as ethnicity or health comorbidities, were lacking. This shortfall restricted analysis depth, especially in understanding how demographic factors and pre-existing health conditions may influence malpractice litigation outcomes. The lack of such data highlights the need for more comprehensive data collection to inform equitable healthcare policies and practices. Socioeconomic disparities, access to healthcare, and patient-provider communication dynamics might pose barriers in pursuing litigation and thus our results may overlook potential patterns or discrepancies in litigation numbers.

In summary, our analysis of 62 OT-related legal cases, spanning 35 years and 19 states sheds light on the prevailing trends, outcomes, and critical factors influencing litigation in this area, but also reveals the inherent limitations of relying solely on public databases. Notably, our findings underscore the predominance of surgical malpractice claims, with deceased patients' cases resulting in higher average compensation amounts compared to those involving living patients. Despite plaintiffs commonly initiating these claims, defendants—typically healthcare providers or institutions—more often prevailed. This trend highlights the complexity of proving negligence and the possible influence of well-prepared defense strategies. The intricate relationship between donor-related issues and litigation outcomes was also evident, pointing to the critical role of thorough donor assessment and transparent communication with patients. To mitigate the expected anticipation of increased litigation numbers in the future and enhance patient care, the transplantation field must embrace the lessons learned from historical cases, fostering a culture of collaboration and trust along with comprehensive patient support.

## Data Availability

The data analyzed in this study is subject to the following licenses/restrictions: Verdictsearch Legal Database is a subsidiary of ALM Media. The data utilized in this study are proprietary to ALM Media, and as such, they are subject to ALM Media usage restrictions. These restrictions include limitations on reproduction, distribution, and public sharing of the data. Requests to access these datasets should be directed to Panos Arvanitis, panagiotis_arvanitis@brown.edu.
